# Evaluation and Treatment of a Ureterosciatic Hernia Causing Hydronephrosis and Renal Colic

**DOI:** 10.1089/cren.2015.29005.sal

**Published:** 2015-10-01

**Authors:** Keyan Salari, Emily M. Yura, Mukesh Harisinghani, Brian H. Eisner

**Affiliations:** Department of Urology, Massachusetts General Hospital, Harvard Medical School, Boston, Massachusetts.

## Abstract

An 87-year-old female presented with complaints of intermittent severe right renal colic. CT imaging demonstrated a ureterosciatic hernia and moderate hydronephrosis proximal to the portion of the ureter that was herniated through the sciatic foramen. A retrograde pyelogram demonstrated a transition point in the ureter at the location of the hernia. A ureteral stent was placed resulting in straightening of the ureter, resolution of hydronephrosis, and complete resolution of the patient's symptoms.

## Clinical History and Physical Examination

Ureteral herniation is a rare cause of ureteral obstruction and typically involves the inguinal, femoral, and obturator regions.^1^ Ureterosciatic hernia (USH) is the rarest form of ureteral herniation, with fewer than 30 cases reported in the literature since 1947.^2^ The greater sciatic foramen is a potential space mostly occupied by the piriformis muscle, and sciatic hernias are thought to principally result from a defect in the parietal pelvic fascia or atrophy of the piriformis muscle. An 87-year-old woman presented with intermittent bothersome right renal colic. The bouts of pain occurred weekly over the 4 months before evaluation and typically lasted 2 to 3 days in duration. Pertinent history included a hysterectomy at age 63 and an exploratory laparotomy with lysis of adhesions for the treatment of a small bowel obstruction at age 84. Physical examination was notable for a body mass index of 14.4 kg/m^2^ and was otherwise unremarkable.

## Diagnostic Studies

**FIG. 1.**
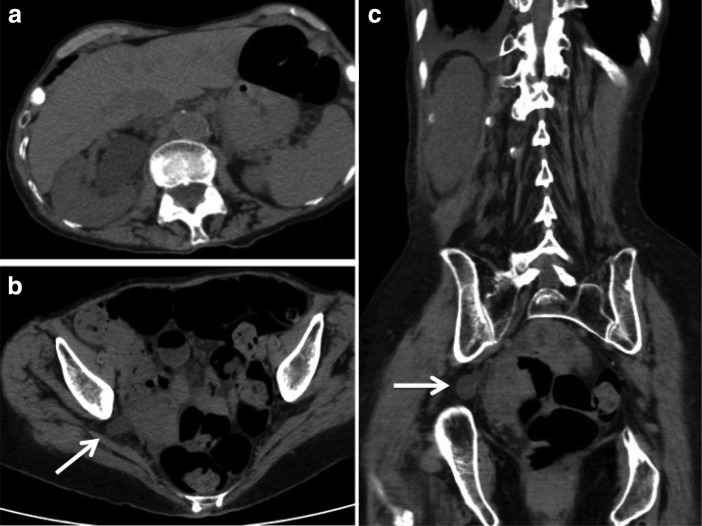
CT scan demonstrating hydroureteronephrosis secondary to herniation of the right ureter into the greater sciatic foramen. **(a)** Shows hydronephrotic renal pelvis and collecting system and **(b, c)** demonstrate the dilated ureter within the greater sciatic foramen (marked by *arrow*).

Serum electrolytes were normal, and the estimated glomerular filtration rate was >60 mL/minute. A CT scan demonstrated hydronephrosis extending from the portion of the ureter that was herniated through the greater sciatic foramen and extending to the proximal ureter and kidney. Axial and coronal CT images are shown in Figure 1, demonstrating hydronephrosis (Fig. 1a), and a dilated ureter herniated through the greater sciatic foramen marked with a white arrow (Fig. 1b, c).

## Intervention and Outcome

**FIG. 2.**
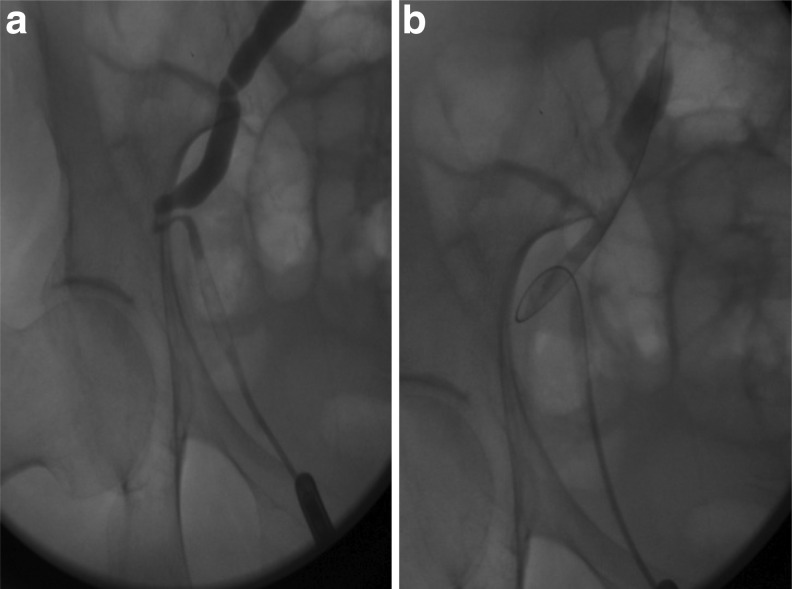
Retrograde pyelogram demonstrating dilation of the ureter proximal to the area of herniation **(a)** and guidewire bypassing the obstruction and passed into the proximal ureter **(b)**.

**FIG. 3.**
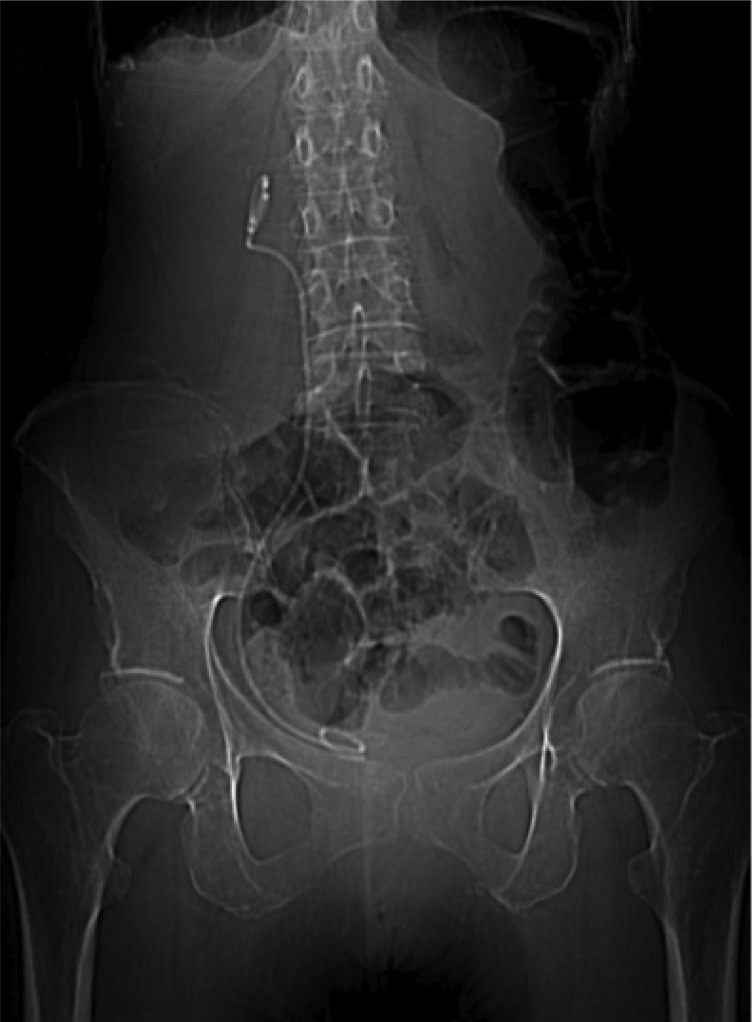
Plain abdominal radiograph demonstrating a right ureteral stent in proper position and a straightened ureter.

A retrograde pyelogram was performed in the operating room and a ureteral stent was placed, which straightened the ureter in the hopes of relieving the intermittent obstruction. Retrograde pyelography demonstrating the position of the USH and proximal hydroureteronephrosis is shown in Figure 2a, and placement of a guidewire is shown in Figure 2b. The patient was seen 1 month after stent placement and reported complete resolution of her symptoms. Figure 3 is a plain abdominal radiograph, which demonstrates that ureteral stent placement straightened the ureter and likely has moved the ureter into a more typical position medial to the sciatic foramen. The patient was offered minimally invasive surgical correction, but has chosen to defer definitive treatment in favor of serial stent changes. To date, she has been followed for 1 year since the first stent placement, with two additional stent changes and no recurrence of symptoms.

## Abbreviations Used

CT: computed tomography
USH: ureterosciatic hernia
